# A Case of Hepatic Glomerulosclerosis with Monoclonal IgA1-*κ* Deposits

**DOI:** 10.1155/2018/4748357

**Published:** 2018-10-01

**Authors:** Yusuke Okabayashi, Nobuo Tsuboi, Naoko Nakaosa, Kotaro Haruhara, Go Kanzaki, Kentaro Koike, Akihiro Shimizu, Akira Fukui, Hideo Okonogi, Yoichi Miyazaki, Tetsuya Kawamura, Makoto Ogura, Akira Shimizu, Takashi Yokoo

**Affiliations:** ^1^Division of Nephrology and Hypertension, Department of Internal Medicine, The Jikei University School of Medicine, Tokyo, Japan; ^2^Department of Analytic Human Pathology, Nippon Medical School, Tokyo, Japan

## Abstract

Glomerular immunoglobulin A (IgA) deposition is a common finding in hepatic glomerulosclerosis; thus, this disease is also called hepatic IgA nephropathy. However, only a small number of patients with hepatic IgA nephropathy have active glomerular lesions, so functional decline is slow in most cases. In this report, we describe a 60-year-old man who developed nephrotic syndrome and progressive renal impairment during follow-up for alcoholic liver cirrhosis. A renal biopsy showed a membranoproliferative glomerulonephritis-like pattern; diffuse double-contours of the glomerular basement membrane and focal active glomerular lesions with moderate-to-severe endocapillary proliferation and fibrocellular crescents. Immunofluorescence findings revealed granular staining for monoclonal IgA1-*κ* and C3 on the peripheral capillary walls. Laboratory examinations did not reveal any definitive evidence of myeloproliferative disorders. Therefore, this case may represent a previously unrecognized etiology of renal injury in relation to liver cirrhosis that is characterized by monoclonal IgA1-*κ* deposits and proliferative glomerulonephritis.

## 1. Introduction

Hepatic glomerulosclerosis is a form of glomerulopathy found in patients with liver cirrhosis [1–3]. The histopathological findings of hepatic glomerulosclerosis include increased mesangial matrices, subendothelial and paramesangial deposits, and diffuse double-contours in the glomerular basement membrane. Reflecting the ischemic changes associated with hepatorenal syndrome, hepatic glomerulosclerosis is typically accompanied by relatively advanced chronic lesions such as glomerulosclerosis, interstitial fibrosis, and renal tubular atrophy [1–3]. Because this disease state is often accompanied by immunoglobulin A (IgA) deposits in glomeruli, it is also termed hepatic IgA nephropathy. Although liver injury and/or a portal venous shunt may cause significant loss of IgA clearance from the liver, the pathogenesis of this disease is not well understood [4–6]. Unlike patients with primary IgA nephropathy, hepatic glomerulosclerosis cases with a large amount of proteinuria or active glomerular lesions are rare [1–3].

We report an atypical case of hepatic glomerulosclerosis showing progressive loss of renal function together with nephrotic syndrome and active glomerular lesions. To our knowledge, this is the first case report of hepatic glomerulosclerosis with monoclonal IgA-*κ* deposits in the glomeruli.

## 2. Case Report

The patient was a 60-year-old man who was diagnosed with alcoholic liver cirrhosis and type 2 diabetes when he was 50 years old. His drinking history was 540–720 mL Japanese sake per day for 35 years, and his smoking history was 30 cigarettes per day for 30 years. His ascites increased in 2008 when he was 57 years old, and he repeatedly exhibited symptoms of hepatic encephalopathy. Due to the liver cirrhosis symptoms, the patient was treated with several medications including furosemide, spironolactone, lactulose, and total amino acid preparation. To prevent the complications of liver cirrhosis, coil embolization to a portal venous shunt was performed twice. The patient was admitted to our hospital in June 2012 because of slowly progressive renal impairment and nephrotic syndrome.

Upon admission, the patient's height and weight were 166 cm and 64 kg, respectively. His body temperature was 36.7°C and his blood pressure was 150/60 mmHg. His consciousness was clear. His abdomen was slightly expanded but exhibited no tenderness. The liver and spleen were not palpable. No rash or purpura was noted on the skin. Diabetic and/or hypertensive changes were not observed in the ocular fundus.

The laboratory findings on admission were hemoglobin level of 9.4 g/dL (normal range 13.5–17.6 g/dL), platelet count of 11x10^4^/*μ*L (normal range, 13.1–36.2x10^4^/*μ*L), prothrombin time measurement of 68% (normal range, 70–130%), total bilirubin level of 0.9 mg/dL (normal range, 0.3–1.2 mg/dL), NH_3_ level of 85 mg/dL (normal range, 30–80 mg/dL), blood urea nitrogen level of 41 mg/dL (normal range, 8–20 mg/dL), serum creatinine concentration of 1.77 mg/dL (normal range, 0.5–1.1 mg/dL), serum total protein level of 5.7 g/dL (normal range, 6.7–8.3 g/dL), serum albumin level of 2.1 g/dL (normal range, 3.5–5.2 g/dL), total cholesterol of 188 mg/dL (normal range, 120–219 mg/dL), and HbA1c of 5.8% (normal range, 4.3–5.8%). The serum levels of IgG were 1558 mg/dL (normal range, 870–1700 mg/dL), of IgA were 481 mg/dL (normal range, 110–410 mg/dL), of IgA1 were 398 mg/dL (normal range, 50–314 mg/dL), of IgA2 were 83 mg/dL (normal range, 10–156 mg/dL), and of IgM were 219 mg/dL (normal range, 35–220 mg/dL). The serum levels of free *κ* and *λ* light chains were 149.0 mg/L (normal range, 3.3–19.4 mg/L) and 106.0 mg/L (normal range, 5.7–26.3 mg/L). The serum free light chain ratio was within normal range. The serum level of complement factor C3 was 79 mg/dL (normal range, 65–135 mg/dL), of C4 was 17 mg/dL (normal range, 13–35 mg/dL), and of CH50 was 41.1 U/mL (normal range, 30–50 U/mL). All of the other serology findings including anti-nuclear antibody, hepatitis B virus surface antigen, hepatitis C virus antibody, anti-neutrophil cytoplasmic antibody, and anti-glomerular basement membrane antibody were negative. There was no M-spike on serum and urine protein electrophoresis. A serum test for a cryoglobulin precipitation was negative.

The urinary sediments showed many red blood cells in high power fields together with granular casts and dysmorphic red blood cells. The urinary protein excretion was 4.7 g/day. The 24-hour creatinine clearance was 45 mL/min. Computed tomography revealed liver deformity with moderate accumulation of ascites. The kidneys were normal in size and there were no signs of urinary tract obstruction.

**Figure 1:**
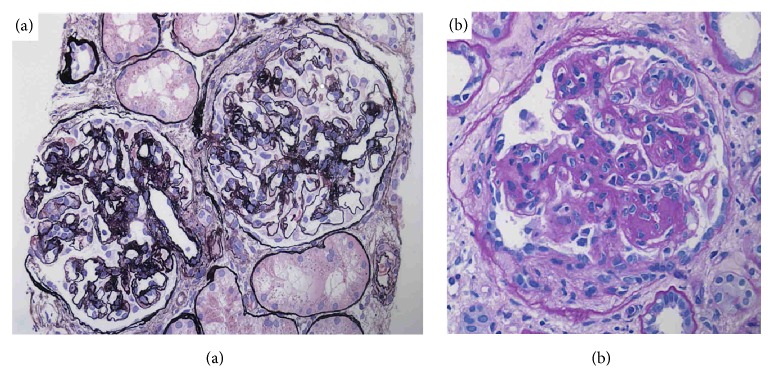
Light microscopy findings. In the renal biopsy specimen, glomeruli showed diffuse double-contours in the glomerular basement membrane and moderate to severe focal endocapillary and mesangial hypercellularity (a). Some nonsclerotic glomeruli were accompanied by fibrocellular crescents (b) ((a), periodic acid methenamine silver stain, original magnification 400×; (b), periodic acid-Schiff stain, original magnification 400×).

**Figure 2:**
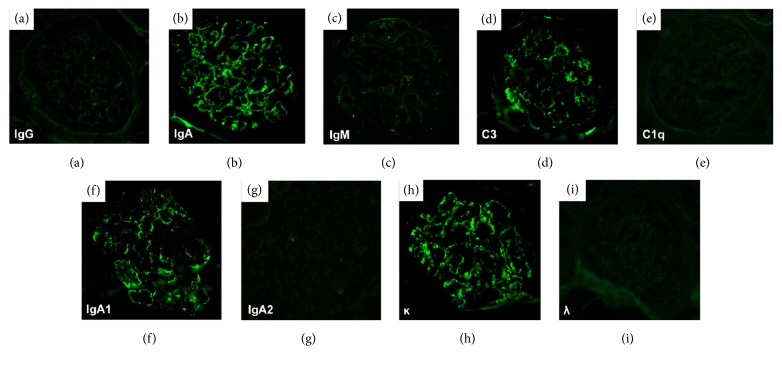
Immunofluorescence microscopy findings. Fluorescent immunostaining showed positive staining for IgA and C3, whereas IgG, IgM, and C1q staining were negative. Fluorescent immunostaining of IgA subtypes and light chains showed positive staining for IgA1 and *κ* light chains, while staining for IgA2 and *λ* light chains was negative.

**Figure 3:**
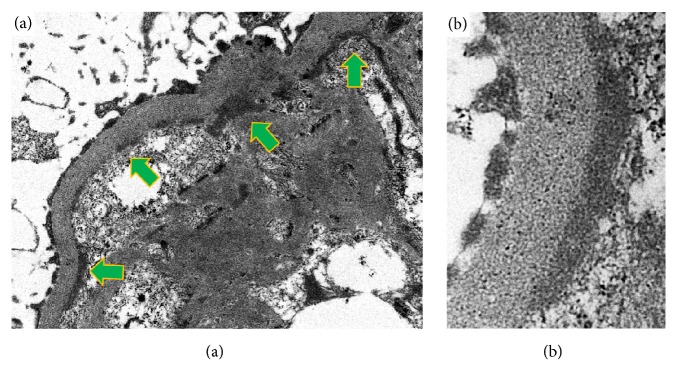
Electron microscopy findings. Electron microscopic examination showed nonorganized deposits in the subendothelial area along the glomerular basement membrane and paramesangial area ((a), original magnification 12,000×; (b), original magnification 50,000×). Because no glomeruli were identified in the portion of the biopsy specimen fixed in glutaraldehyde, the formalin-fixed and paraffin-embedded specimen was reprocessed for electron microscopy. Arrows indicate nonorganized deposits.

The renal biopsy specimens contained a total 28 glomeruli, 12 of which were globally sclerotic. The degree of interstitial fibrosis/tubular atrophy was 50–60% of the total biopsy specimen identified. Moderate fibrous intimal hyperplasia was observed in the arcuate artery. Diffuse segmental double-contours of the glomerular basement membrane and mesangial cell hypercellularity were identified in nonsclerotic glomeruli, exhibiting a membranoproliferative glomerulonephritis-like pattern (Figure 1(a)). Some glomeruli showed moderate-to-severe endocapillary hypercellularity, accompanied by fibrocellular crescents (Figure 1(b)). Fluorescent immunostaining showed granular staining of IgA and C3, but not of IgG, IgM, or C1q, on glomerular capillaries and some mesangial areas (Figures 2(a)–2(e)). Among the IgA subtypes, staining of IgA1 (GenWay Biotech, San Diego, CA, USA) was observed, but staining of IgA2 (GenWay Biotech) was not identified (Figures 2(f) and 2(g)). With light chain immunostaining, only *κ* (SouthernBiotech, Birmingham, AL, USA) was identified and no *λ* staining (SouthernBiotech) was seen (Figures 2(h) and 2(i)). On electron microscopy, the glomerular capillary walls showed double contours. Electron-dense deposits were found in the paramesangium and around the subendothelial space of the glomeruli (Figure 3(a)). No organized structure deposits were identified (Figure 3(b)). Based on these findings, this case was histologically diagnosed as diffuse membranoproliferative glomerulonephritis with monoclonal IgA1-*κ* deposits.

Because this case was accompanied by moderately advanced decompensated liver cirrhosis, there was a concern that the patient may have serious side effects due to aggressive treatment such as the administration of corticosteroids. Thus, supportive treatment based on medications such as RAS inhibitors/diuretics, in addition to dietary therapy including salt restriction/branched-chain amino acid administration, was selected. Although these treatments led to a modest decrease in the urinary protein excretion, the patient's renal dysfunction slowly progressed and finally resulted in end-stage renal failure and initiation of dialysis therapy.

## 3. Discussion

Proteinuria and hematuria are found in about 9% of cases of liver cirrhosis, whereas the frequency of nephrotic syndrome is estimated to be about 1.6% [1–3]. An autopsy study has shown that a moderate or high numbers of glomerular lesions are present in about 50% of liver cirrhosis patients [1]. Interestingly, this patient showed typical features of hepatic glomerulosclerosis, including chronic sclerotic and fibrotic changes, upon histological examination. However, this case was also atypical due to the presence of nephrotic syndrome, progressive renal deterioration, and active glomerular lesions along with depositions of monoclonal IgA1-*κ* in the glomeruli.

The serum increase of IgA1 observed in this case was consistent with what is generally found in hepatic glomerulosclerosis [3]. To our knowledge, however, no previous study has characterized glomerular IgA immunocomplexes in hepatic glomerulosclerosis. Therefore, this case may represent a previously unrecognized etiology of renal injury related to liver cirrhosis that is characterized by monoclonal IgA1-*κ* deposits and proliferative glomerulonephritis.

There have been some reported cases of glomerulonephritis characterized by monoclonal IgA glomerular deposits [7–12]. The six reported cases and our case occurred with or without clinically apparent myeloproliferative disorders, such as multiple myeloma, monoclonal gammopathy of undetermined significance, or dysproteinemia. Although bone marrow puncture was not performed in our case, no abnormalities in protein electrophoresis of serum and urine or the serum free light chain ratio were observed; therefore, the involvement of myeloproliferative diseases was excluded. In addition, our patient did not have a clinical history or show serological evidence of viral infection or autoimmune disorders. Therefore, these results suggest that factors other than paraprotein diseases, viral infections, or autoimmune diseases are involved in the pathogenesis of glomerulopathy associated with monoclonal IgA1-*κ* deposits.

Similar to patients with idiopathic IgA nephropathy, recent studies on the glycosylation of serum IgA in patients with hepatic glomerulosclerosis identified abnormalities in the IgA hinge lesion (increase in galactosylation, decrease in sialylation), which indicates that abnormal glycosylation in the IgA hinge lesion may result in glomerular IgA deposition [3]. One study on glomerular light chains in 65 patients with primary IgA nephropathy, but without other potentially contributing diseases, revealed that six cases (9.2%) had monoclonal IgA deposits (5 IgA-*λ* type cases, 1 IgA-*κ* case) [13]. However, there was no clear difference in the clinicopathological findings and renal outcomes between the patients with monoclonal IgA deposits and those with polyclonal IgA deposits. Therefore, the pathological significance of monoclonal IgA glomerular deposits is undetermined, even in patients diagnosed with primary IgA nephropathy without background diseases such as liver cirrhosis.

There are no specific treatments for hepatic glomerulosclerosis, and the degree of liver disease is an important predictor of the progression of end-stage renal disease (ESRD). Babbs et al. reported that surgery for portal hypertension resulted in remission of the nephrotic syndrome in a patient with hepatic glomerulosclerosis [14]. In our current case, however, treatment of portal hypertension, such as coil embolization of a portal venous shunt, had no effect on the renal outcome. Considering their adverse events, we did not choose corticosteroid therapy in our case. In comparison, Takeda et al. reported two patients with hepatic glomerulosclerosis who were successfully treated with corticosteroids with no adverse events [15]. Further reports and studies are needed to clarify the clinical utility of corticosteroid therapy for hepatic glomerulosclerosis.

In conclusion, this case suggests that a hepatic glomerulosclerosis subgroup defined by monoclonal deposits of glomerular IgA and active glomerular lesions may exist. The pathogenesis of monoclonal IgA deposits in the glomeruli in this case has yet to be determined. Thus, further studies to evaluate immunoglobulin subclasses and light chain staining in similar cases are needed to elucidate the pathogenesis of this previously unrecognized renal injury etiology in patients with liver cirrhosis.
